# Dermatomyositis With Anti-MDA5 Antibodies: Bioclinical Features, Pathogenesis and Emerging Therapies

**DOI:** 10.3389/fimmu.2021.773352

**Published:** 2021-10-20

**Authors:** Anaïs Nombel, Nicole Fabien, Frédéric Coutant

**Affiliations:** ^1^ Immunology Department, Lyon-Sud Hospital, Hospices Civils de Lyon, Pierre-Bénite, France; ^2^ Immunogenomics and Inflammation Research Team, University of Lyon, Edouard Herriot Hospital, Lyon, France

**Keywords:** myositis, dermatomyositis, idiopathic inflammatory myopathies, MDA5, COVID-19, SARS-CoV-2, autoantibodies, autoantibody

## Abstract

Anti-MDA5 dermatomyositis is a rare systemic autoimmune disease, historically described in Japanese patients with clinically amyopathic dermatomyositis and life-threatening rapidly progressive interstitial lung disease. Subsequently, the complete clinical spectrum of the disease was enriched by skin, articular and vascular manifestations. Depending on the predominance of these symptoms, three distinct clinical phenotypes with different prognosis are now defined. To date, the only known molecular component shared by the three entities are specific antibodies targeting MDA5, a cytosolic protein essential for antiviral host immune responses. Several biological tools have emerged to detect these antibodies, with drawbacks and limitations for each of them. However, the identification of this highly specific serological marker of the disease raises the question of its role in the pathogenesis. Although current knowledge on the pathogenic mechanisms that take place in the disease are still in their enfancy, several lines of evidence support a central role of interferon-mediated vasculopathy in the development of skin and lung lesions, as well as a possible pathogenic involvement of anti-MDA5 antibodies. Here, we review the clinical and biological evidences in favor of these hypothesis, and we discuss the contribution of emerging therapies that shed some light on the pathogenesis of the disease.

## Introduction

The idiopathic inflammatory myopathies (IIM) are a heterogeneous group of rare connective tissue diseases, characterized by inflammation of several organs and tissues other than the muscles, such as the skin and the lungs. IIM include necrotizing immune-mediated myositis, inclusion body myositis, antisynthetase syndrome and dermatomyositis (DM) (1). These four subgroups are very heterogeneous in their clinical, prognostic and pathological features, which renders the diagnosis and the treatment challenging. However, the discovery and the inclusion of myositis specific autoantibodies (MSA) in the diagnostic algorithm of myositis allowed a better definition of subgroups of patients in terms of clinical phenotypes, prognosis and response to treatment. One of these MSA, the anti-melanoma differentiation-associated gene 5 (MDA5) antibodies (Abs), was originally identified in a specific subset of DM, named clinically amyopathic DM (CADM), associated with an increased risk for rapidly progressive interstitial lung disease (RP-ILD). The clinical presentation of anti-MDA5 DM differs substantially from the other forms of DM, with three distinct clinical phenotypes, according to the predominance of pulmonary, skin-articular or vascular symptoms (2). The pathogenesis of these three forms of anti-MDA5 DM is largely unknown, and to date, the only common molecular characteristic of these entities is the presence in the blood of the patients of Abs targeting the antigen MDA5, a highly specific biomarker of the disease, sometimes difficult to detect with usual techniques. MDA5 is a cytosolic protein, essential for antiviral host immune responses, which functions as a virus RNA sensor and induces, once activated, the production of type I interferons (IFN-I) and pro-inflammatory cytokines by the cell. The pathogenic role of anti-MDA5 Abs is currently unknown, but its involvement in the disease by targeting a critical actor of the immune system could be congruent with the concept of autoimmunity induced by infectious agents.

In this review, we outline the clinical phenotypes of the DM with anti-MDA5 Abs, the distribution and the functions of MDA5, as well as the biological tools available for the detection of anti-MDA5 Abs and their limitations. We focus on recent biological data that provide insight into the pathogenesis of the disease, and we propose a pathophysiological model centered on vascular dysfunction and dysregulated immune system. In this proposed model, we will discuss whether the Abs specific of the disease could be critical players in disease pathogenesis, and not just biomarkers.

## Clinical Spectrum in Adults of the Dermatomyositis With Anti-MDA5 Antibodies

Anti-MDA5 DM is a rare disease representing less than 2% of IIM in Europe (3). Among the subgroup of DM, the prevalence of anti-MDA5 DM ranges from 7 to 60%, with higher prevalence in Asian (11-60%) than in Caucasian (7-16%) (**Table 1**) (1, 4–19). Similar to other autoimmune diseases, anti-MDA5 DM occurs mainly in women, with a female/male ratio that ranges from 0.6 to 7.3 (F/M >1 in 14 out of 16 studies) (**Table 1**) (4–19).

**Table 1:** Ethnicity and prevalence of dermatomyositis with anti-MDA5 antibodies.

Country	DM cases* (n)	Anti-MDA5^+^	Detection technique	CADM among anti-MDA5^+^	CADM cases (n)	Anti-MDA5^+^ among CADM	Prevalence of women anti-MDA5^+^	ILD among anti-MDA5^+^	RP-ILD among anti-MDA5^+^	Ref	Publication year
Japan	42	19%	IP/IB/IIF	100%	15	53%	75%	88%	50%	(4)	2005
Japan	30	27%	IP	75%	13	46%	88%	100%	100%	(5)	2009
Japan	65	22%	IP/ELISA	57%	15	53%	79%	100%	71%	(6)	2010
Japan	37	35%	IP/IB	85%	15	73%	69%	92%	54%	(7)	2010
Japan	55	11%	IP	17%	ND	ND	50%	83%	50%	(8)	2011
Japan	376	11%	IP/IB/ELISA	77%	51	65%	79%	93%	65%	(9)	2011
Japan	79	22%	IP/ELISA	82%	21	67%	88%	94%	71%	(10)	2012
China	84	23%	ELISA	26%	8	63%	58%	ND	79%	(11)	2012
China	64	23%	ELISA	80%	32	38%	60%	100%	60%	(12)	2012
China	43	60%	ELISA	35%	9	100%	39%	100%	39%	(13)	2013
China	213	21%	ID	77%	81	42%	71%	82%	64%	(14)	2020
North America	77	13%	IP	50%	ND	ND	88%	67%	20%	(15)	2011
North America	160	7%	IP	46%	ND	ND	73%	73%	ND	(16)	2013
Spain	117	12%	ELISA/IB/IIF	57%	15	53%	64%	64%	57%	(17)	2014
Italy	34	15%	IP-WB/ELISA/IIF	100%	ND	ND	60%	60%	20%	(18)	2014
Brazil	131	16%	ELISA	24%	22	23%	71%	38%	ND	(19)	2018
France	54	9%	IIF/ID	ND	ND	ND	ND	ND	ND	(1)	2018

*Selection of cohorts with at least 30 patients (cohorts with JDM patients excluded).

DM, dermatomyositis; CADM, clinically amyopathic dermatomyositis; JDM, juvenile dermatomyositis; ILD, interstitial lung disease; RP-ILD, rapidly progressive interstitial lung disease; IP, immunoprecipitation; IB, immunoblot; IIF, indirect immunofluorescence; ELISA, enzyme-linked immunosorbent assay; WB, western blot; ID, immunodot assay; ND, not done.

Systemic autoimmune DM are characterized by skin manifestations accompanying or preceding muscle weakness, and, to various extents, lung lesions. The DM associated with anti-MDA5 Abs shares clinical features with DM but also has disease-specific traits. Indeed, the patients with anti-MDA5 DM may have the hallmark cutaneous manifestations of DM, but the disease is also associated with specific skin manifestations. Moreover, the muscle disease is minimal or absent, and pulmonary interstitial lesions may be rapidly progressive which is not the case in other forms of DM.

### Muscular Manifestations of Anti-MDA5 Dermatomyositis

Anti-MDA5 DM was first described by Sato et al. in 2005, in a Japanese cohort with CADM and RP-ILD (4). As defined by Sontheimer et al. (20), CADM patients display the hallmark cutaneous manifestations of DM for at least 6 months, without muscle weakness. The hypomyopathic forms of CADM are associated with elevated muscle enzymes and/or abnormalities in EMG or muscle biopsy, whereas amyopathic DM patients have the cutaneous manifestations of DM for at least 6 months, without clinical or biological signs of muscle damage. However, CADM is not sufficient to define anti-MDA5 DM, as not all CADM patients have anti-MDA5 Abs. The proportion of anti-MDA5 Abs positive patients among CADM ranges from 23% to 100%, depending on the cohorts (**Table 1**) (4–7, 9–14, 17, 19). Conversely, the prevalence of CADM among anti-MDA5 Abs positive patients is also very inconsistent (17 to 100%, **Table 1**) (4–19).

When present, muscle damage mainly affects proximal muscles (2, 12). Several histological features of muscle biopsies are shared by different forms of DM, and include perifascicular fiber atrophy, perivascular inflammation, infiltrates of T and B cells, diffuse class I major histocompatibility complex (MHC-I) expression and deposition of complement attack complex (21, 22). In contrast, muscle biopsies of patients with anti-MDA5 DM are often normal or highlight only rare anomalies. Inflammation is absent or scarce and cellular infiltrates, mostly macrophages, cluster focally in the perimysium. MHC-I expression is focal or absent. Complement membrane attack complex deposition is rarely observed. Furthermore, IFN signature in skeletal muscles of patients with anti-MDA5 DM is up-regulated, compared with healthy subjects but lower than patients with anti-MDA5 negative DM (21, 22). These observations are consistent with the mild muscle phenotype frequently associated with anti-MDA5 DM.

### Mucocutaneous and Articular Manifestations of Anti-MDA5 Dermatomyositis

The hallmark cutaneous manifestations of the DM spectrum occur with similar prevalence in patients with anti-MDA5 DM. They include periorbital heliotrope (blue–purple) rash with edema, erythematous rash on the face, or the anterior chest (in a V-sign), and back and shoulders (in a shawl sign), violaceous papules or plaques located on the dorsal part of the metacarpophalangeal or interphalangeal joints, called Gottron’s papules and cracked palmar fingertips (“mechanic’s hands”). It is important to underline here the danger of potential misdiagnosis of patients with anti-MDA5 DM, given the absence of clinical evidence of myopathy and also sometimes the similarities of DM-specific manifestations, such as Gottron’s papules, with other skin lesions, such as psoriatic lesions (23, 24).

Anti-MDA5 DM is also associated with a more specific cutaneous phenotype, that includes palmar papules and skin ulcerations, reported in both Caucasian (2, 15, 16, 25–28) and Asian populations (9, 10, 12, 29–31). Unlike Gottron’s papules, palmar papules are often located on the palmar surface or lateral sides of the fingers, especially over metacarpophalangeal and interphalangeal joints. Many of these lesions have a central ivory coloration, and they are frequently painful. Palmar papules can be associated with hyperkeratosis, and complicated of ulcerations.

Skin ulcerations associated with anti-MDA5 DM usually manifest as deep painful ulcers localized over Gottron’s papule, involving the digital pulp and nail folds, or over Gottron’s sign on the knees, elbows, or both. The histology of skin ulcerations shows vasculopathy, pauci-inflammatory or characterized by medium vessel wall infiltration with mononuclear cells, and intravascular thrombus (12, 15). Major complications can occur, such as gangrene and osteomyelitis, potentially leading to digital amputation (25, 32, 33).

Less specific auricular skin lesions have also been described in DM with anti-MDA5 Abs, such as antihelix/helix violaceous macules and erythematous auricular papules (34, 35). These particular cutaneous phenotypes, seldom mentioned in the literature, could be clinical markers of poor prognosis, although most previous studies examining auricular skin lesions in the disease have been limited to isolated case reports (36). Other less specific mucocutaneous lesions have been described in DM with anti-MDA5 Abs. They include oral ulcers, panniculitis, alopecia and flagellate erythema (15, 17, 37, 38).

Since 2010, many studies reported the increased prevalence of arthritis (42-82%) and arthralgia in patients with anti-MDA5 DM (2, 9, 15, 16). The arthritis described in the patients closely resembles that found in rheumatoid arthritis, potentially leading to misdiagnosis in the absence of other DM symptoms. They are associated with morning stiffness and are typically symmetric, affecting the small joints of the hands, but also the wrists and the ankles (16, 23, 39). Although rarely described in the literature, the conventional radiography do not show any bone erosion. However, erosions have been exceptionally reported by magnetic resonance imaging (16, 39). When explored, the rheumatoid factor was positive in some cases, but anti-cyclic citrullinated peptide Abs were not detected in any of the patients (27, 39).

Another differential diagnosis of these articular forms of anti-MDA5 DM is the psoriatic arthritis. Indeed arthritis is a common feature associated with psoriasis and some cases of anti-MDA5 DM initially manifest as psoriasiform skin lesions (23, 24, 40). In the literature, one case has been reported of a patient with psoriasiform lesions, associated with severe symmetrical polyarthritis of large and small joints, and a mild proximal weakness in upper and lower extremities. All of these elements initially led to the misdiagnosis of psoriatic arthritis (23). However, thoracic CT scan subsequently showed a bilateral ground-glass pattern, and laboratory tests revealed blood positivity for anti-MDA5 Abs. Thus, the association of arthritis and psoriasiform lesions, associated with weak muscle or pulmonary involvement may lead to a misdiagnosis of psoriatic arthritis, if anti-MDA5 Abs are not explored.

Finally, some patients have a phenotype close to the antisynthetase syndrome (i.e. arthritis, Raynaud’s syndrome, mechanic’s hands, ILD), further complicating the diagnosis (16, 41).

### Lung Manifestations of Anti-MDA5 Dermatomyositis

Anti-MDA5 DM is associated with poor prognosis due to a high prevalence of RP-ILD. ILD is the result of inflammation and fibrosis of the lung parenchyma and specific patterns of ground-glass attenuation are observed on high-resolution computed tomography (42). Significant disparities between association of anti-MDA5 Abs and RP-ILD have been described in the cohorts. For instance, in Japan and in East Asia populations, ILD occurs in 82 to 100% of patients with anti-MDA5 DM, and RP-ILD in 39 to 100% of patients (**Table 1**) (4–14). The incidence of RP-ILD appears less important in Caucasian populations with 38 to 73% of anti-MDA5 DM patients having ILD and 20 to 57% having RP-ILD (**Table 1**) (15–19), some studies reporting a significant association (17, 25) while others do not (15, 16, 19).

It is of importance to note that ILD can be absent at diagnosis and develop many years later, or completely absent in some patients over years of follow-up. Conversely, ILD can sometimes reveals anti-MDA5 DM. For instance, three Japanese patients with ILD and anti-MDA5 Abs, but without cutaneous nor muscular manifestation, had a fatal outcome within two months after onset (43). Similarly, two patients initially hospitalized for fever and dyspnea developed skin symptoms characteristic of DM one month after onset (44).

The mortality rate of patients who develop RP-ILD is reported to be approximately 50%, with most deaths occurring during the very early stages of the illness (6, 7, 9, 10, 13, 43, 45, 46). Some studies suggest that beyond 6 months after onset, disease progression tends to settle down, and relapse seems uncommon (10). However, recurrences have also been described in the form of cutaneous and/or severe respiratory relapse many years after onset and after months of clinical remission and new treatments are needed to contain the exacerbation (47, 48).

### Association of Anti-MDA5 Dermatomyositis With Malignancy

Several DM are associated with an increased risk for cancer, such as DM with anti-TIF1γ Abs, and to a lesser extent, DM with anti-NXP2 Abs (3, 49). By contrast, the association of anti-MDA5 DM with cancer has been explored in large cohorts, without any correlation reported (7, 9, 10, 12). However, few cases of cancer have been reported in patients with anti-MDA5 DM (33, 37, 50). Metastatic small cell carcinoma with liver involvement was detected 12 months after anti-MDA5 DM diagnosis in a 60 year-old French woman (50). Another study presented the case of a man diagnosed simultaneously with both anti-MDA5 DM and thyroid cancer (37). Even if no evident causal link is established, these few cases highlight that anti-MDA5 DM and cancer might not be exclusive.

### Other Clinical and Biological Features of Anti-MDA5 Dermatomyositis

Among the constitutional symptoms, the fever has been described in up to 74% of anti-MDA5 DM at onset [33-74%] (7–9, 11, 13, 16). Several distinctive features in blood tests are also reported in the disease, such as elevated ferritinemia, with no significant elevation of C-reactive protein. Ferritin levels correlate with the severity of the disease and ILD (6, 7, 51, 52). Liver dysfunction is also recurrently observed in anti-MDA5 DM, as evidenced by elevated levels of alanine transaminase or gamma-glutamyl transferase, without elevated creatine kinase (6, 7, 21, 53). Liver biopsies show steatosis and hepatocyte ballooning (53). Furthermore, liver enzymes might increase as ferritinemia increases and ILD worsens (7). Decreased CD4^+^ and CD8^+^ T cell counts and a raised CD4^+^/CD8^+^ ratio are frequently described in peripheral blood of patients with anti-MDA5 DM, even before immunosuppressive treatment (11, 54). When studying the relation between pulmonary lesions and lymphopenia, an increase of CD4^+^ and CD8^+^ T cell counts is observed in parallel with pulmonary lesions improvement after treatment. Inversely, CD4^+^ and CD8^+^ T cell counts decrease and the CD4^+^/CD8^+^ ratio increases in patients with ILD refractory to treatment (54). Finally, positivity for anti-TRIM21 (Ro52) Abs (55, 56) and older age (14, 29) are indicators of poor prognosis while the female sex and articular form might be factors of good prognosis (57).

### Dermatomyositis With Anti-MDA5 Antibodies: Three Distinct Clinical Phenotypes

**Figure 1:**
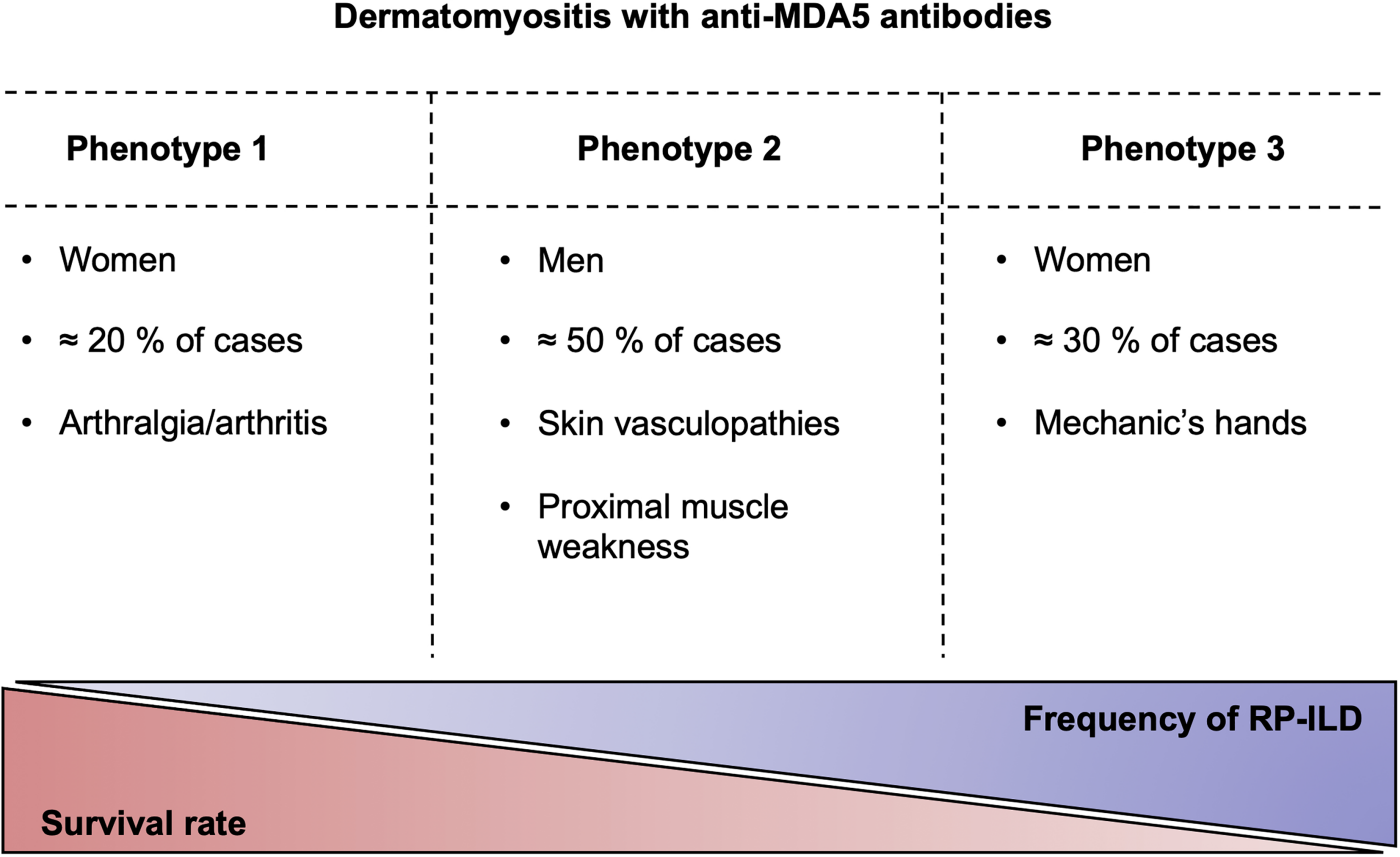
The clinical phenotypes of anti-MDA5 dermatomyositis. Anti-MDA5 dermatomyositis can be divided into three clinical phenotypes with varying degrees of pulmonary damage, which is inversely correlated with the survival. RP-ILD, rapidly progressive interstitial lung disease.

The heterogeneity of clinical features and outcomes among patients with anti-MDA5 DM prompted to divide the clinical spectrum of anti-MDA5 DM into three distinct clinical subgroups (**Figure 1**) (2). The first one is composed mainly of women with RP-ILD associated with mechanic’s hands, with the highest mortality rate (80%). A rheumatologic group (55% of cases) is also made mostly of women with arthralgia or arthritis (83%), less frequent RP-ILD (17%) and a better prognosis. The third clinical phenotype mainly encompasses men with symptoms dominated by skin vasculopathies, including a Raynaud’s phenomenon (82%), skin ulcers (77%), digital necrosis (32%) and calcinosis (23%). This last subgroup is frequently associated with proximal muscles weakness (68%), with relatively few RP-ILD (23%) and it is of intermediate prognosis. This classification was already suggested by a Spanish group in 2014 (17) and could permit an appraisal of the prognosis of patients with anti-MDA5 DM.

## Juvenile Dermatomyositis Associated With Anti-MDA5 Antibodies

Juvenile DM (JDM) is associated with anti-MDA5 Abs in 6 to 12% of cases in European and North American cohorts, and constitutes the third most frequent DM-associated Abs after anti-TIF1γ and anti-NXP2 Abs (56, 58–62). The prevalence of the anti-MDA5 Abs is higher in Japanese cohorts, with 24 to 38% of patients having anti-MDA5 Abs associated JDM (63–65). The anti-MDA5 JDM phenotype is associated with a higher frequency of constitutional symptoms (weight loss, fever and adenopathy) and milder muscle involvement compared to the other forms of JDM (58, 61, 62, 65).

Similarly to the adult form, the specific cutaneous phenotype found in anti-MDA5 DM, i.e. the palmar papules and skin ulcerations, are also observed in anti-MDA5 JDM, as well as arthritis, which is more frequent than in other subtypes of JDM (58, 59, 61, 64). These lesions distinguish anti-MDA5 JDM from the other forms of JDM. The arthritis associated with the juvenile form is similar to the one observed in adults, characterized by symmetrical pain and swelling of the small joints of the hands (58, 66). Anti-MDA5 JDM is also associated with an increased risk of ILD (58, 61, 63–65, 67), which is more frequent compared with other forms of JDM associated with MSA, except for anti-synthetase Abs-associated JDM. RP-ILD appears to be less common (<10%) in Caucasian anti-MDA5 JDM, compared to the adult form (58). Thus, a major difference between the juvenile form and the adult form of the disease in Caucasian patients is observed in terms of outcome, since anti-MDA5 JDM has comparable outcome with the other forms of JDM, but flares are less frequent in this subset of JDM and necessitates less medication (58, 59, 61). The observations on the association between anti-MDA5 Abs JDM and RP-ILD are more contradictory in the Japanese population. Indeed, although some studies report a much higher frequency of RP-ILD in Japanese anti-MDA5 JDM patients associated with a poor prognosis, a recent multicenter study reported only 19% of RP-ILD in a cohort of 31 anti-MDA5 Abs positive patients, with a lower rate of relapse than other MSA positive patients (63, 67, 68).

## MDA5 and Anti-MDA5 Antibodies

In 2005, Sato et al. reported that Abs found in the sera of patients with CADM react with a cytoplasmic protein of 140 kDa (4). In 2009, the same team described MDA5 as the antigenic target of 140 kDa which is recognized by the Abs found in patients with CADM (69).

MDA5 was initially identified in 2002, as a type I IFN-inducible gene in human melanoma cells, and the first function described for this protein was to induce the death of cancer cells (70). Since this first description, MDA5 is now considered as a key sensor of viral infection, mediating the production by the infected cell of IFN-I and the induction of other genes that collectively establish an antiviral host response. MDA5 is encoded by the gene *IFIH1* (interferon-induced with helicase C domain 1) and is part of the Retinoic acid Inducible Gene-I-like receptor family that detect molecular patterns of viruses that have gained access to the cytosol of the infected cell. More specifically, MDA5 senses preferentially long (> 300 bp) RNA-RNA strand pairs, which are mainly RNA viruses replicative intermediates, although some DNA viruses also produce them during their life cycle (71, 72). MDA5 senses these double-stranded RNA (dsRNA) *via* its RNA helicase domain and a C-terminal domain (CTD), and subsequently transmits a signal *via* its homotypic interacting caspase recruitment domain (CARD). Upon recognition of dsRNA, MDA5 assembles into a filament along the dsRNA axis and adopts a ring-like conformation around dsRNA, allowing MDA5 to bind with a strong affinity to its ligand. MDA5 then interacts with the mitochondrial antiviral signaling protein (MAVS), present on the outer membrane of the mitochondria, peroxisomes and other mitochondria-associated membranes. The interaction between MDA5 and MAVS through their respective CARD leads to the activation of the transcription factors interferon regulatory factor 3 and 7 (IRF3 and IRF7), as well as nuclear factor-kappa B (NF-κB). Phosphorylated IRF3, IRF7 and NF-κB then accumulate in the nuclei where they activate antiviral gene transcription, including IFN-I genes, leading to the production of type I IFN (IFNα and IFNβ) and pro-inflammatory cytokines (73, 74).

By this way, MDA5 is able to detect and limit the replication of several RNA viruses of the picornavirus, flavivirus or coronavirus families, as well as DNA viruses of the herpesvirus family for instance (71, 75). But MDA5 is also able to sense endogenous dsRNA, such as mitochondrial dsRNA generated by bilateral transcription of mitochondrial DNA. This biological mechanism is tightly regulated, by mitochondrial degradasome enzymes such as the polynucleotide phosphorylase (PNPase), avoiding the accumulation of dsRNA and thus deleterious activation of MDA5. As a result, PNPase deficiency can cause a massive accumulation of long mitochondrial dsRNA, escaping into the cytosol and leading ultimately to an uncontrolled activation of MDA5 and an upregulation of interferon-stimulated genes (76). In line with this, patients with hypomorphic mutations in *PNPT1*, the gene that encodes for PNPase, have type I interferonopathies characterized by a constitutive activation of the IFN-I axis (77).

### Tissue Distribution and Cellular Location of MDA5

A greater knowledge of the tissue distribution and cellular location of MDA5 is essential to better understand the pathophysiology of anti-MDA5 DM. MDA5 has low tissue specificity in physiological condition (78). A higher expression in the skin and lung tissues of patients with anti-MDA5 DM would be expected compared to expression in muscle tissues. Although studies on MDA5 expression in these target tissues are very rare, MDA5 expression has been shown to be enhanced in skin biopsies of patients with DM, which could offer an element of response to the severe cutaneous symptoms associated with the disease (79).

Defining the cellular location of MDA5 in a pathological context is also critical as pathogenic Abs necessitate to be internalized when the antigenic target is cytosolic, or act by interacting with a protein expressed at the surface of a target cell. At the cellular level, MDA5 was initially described as an intracellular protein found in the cytoplasm of most cells (70). However, Berger et al. studied MDA5 expression and subcellular localization in neutrophils and showed an overexpression of MDA5 both in the cytoplasm and in secretory vesicles as well as a cell surface expression (80). Expression of MDA5 at the surface of other cell types is still being determined. The identification of transient or constitutive expression of MDA5 on the surface of target cells could constitute a critical element in the understanding of the pathogenesis of the disease, in favor of a potential pathogenic role of anti-MDA5 Abs.

### Anti-MDA5 Autoantibodies: The Diagnostic Marker

The identification of the anti-MDA5 Abs in CADM was initially performed by immunoprecipitations (IP) of sera from patients incubated with ^35^S-methionine-labeled K562 cell extracts (4). Since then, radiolabeled IP is considered as the gold standard testing method to detect anti-MDA5 Abs. Although the substitution of radiolabeled antigenic extracts by biotin-labeled recombinant MDA5 constitute a good alternative to bypass the use of radioactive materials (81), IP remains difficult to use in everyday practice, because time-consuming and expensive. Another major issue of IP assay regarding anti-MDA5 Abs identification is the comigration of the MDA5 antigen with other antigens found in DMs, such as the antigens NXP2, TIF1γ and OJ (18, 59, 82). Great expertise is therefore required for correct identification of anti-MDA5 Abs by IP, and alternative interpretation and other assays are therefore required to confirm a serum positivity.

**Figure 2:**
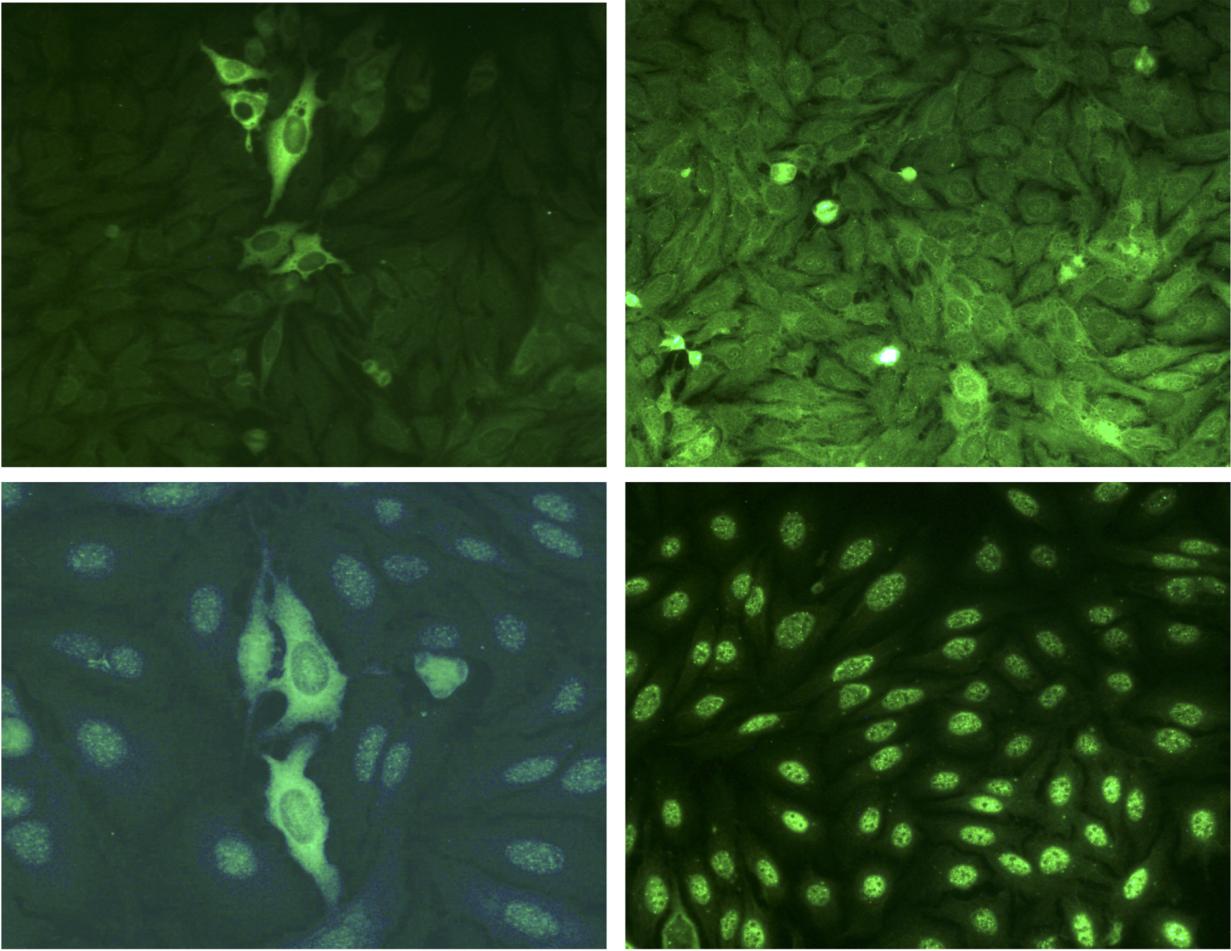
Indirect immunofluorescence patterns of HEp-2 cells stained with anti–MDA5 positive sera. Stainings were performed on HEp-2 cells (Kallestad, Biorad) with sera from patients with anti-MDA5 Abs dermatomyositis. Top left: typical fine granular cytoplasmic staining in rare clustered cells. Top right: granular cytoplasmic pattern in all cells (which can mask the typical cytoplasmic pattern). Bottom left: nuclear speckled pattern, associated with the typical cytoplasmic pattern. Bottom right: isolated nuclear speckled pattern. Note that absence of fluorescence can also be observed with some sera.

For all the reasons mentioned above, IP is used only in a limited number of medical laboratories, which opted now for qualitative or quantitative alternative assays. Qualitative assays include indirect immunofluorescence (IIF) staining on HEp-2 cells and immunodot assays (so-called line blot or dot blot) (83). IIF staining performed on HEp-2 cells with diluted sera from patients with anti-MDA5 DM can give rise to a characteristic cytoplasmic staining with a finely granular appearance, in rare clustered cells and in one study in all cells (**Figure 2**) (4, 84, 85). This difference could be due to the source of HEp-2 cells differentially expressing MDA5 or to the presence of other Abs. However, the scarcity of positive cells generally observed in the microscope field makes the identification of anti-MDA5 Abs by IIF very touchy, and requires a trained eye. Moreover, this IIF pattern is inconstant. In our personal experience that includes a cohort of 31 anti-MDA5 DM, this particular IIF pattern was observed with 50% of sera. Several nonspecific IIF patterns are otherwise observed and have been described, such as a granular cytoplasmic pattern in all cells (which can mask the typical cytoplasmic pattern), or a nuclear speckled pattern, associated or not with the typical cytoplasmic pattern (**Figure 2**) (4). Finally, anti-MDA5 Abs positive sera can also be negative by IIF (17, 18). IIF results should thus always be interpreted by taking in consideration the clinical context and other specific assays should be conducted to confirm a positive IIF pattern or to further explore a negative IIF, if there is a strong clinical suspicion of anti-MDA5 DM.

Commercialized immunodot assays (Line blot LIA, Euroimmun, Lübeck, Germany and dot blot D-Tek, BlueDiver, Mons, Belgium) and particle-based multi-analyte technology (PMAT, Inova, Diagnostics, US) can be used for the detection of anti-MDA5 Abs. These specific qualitative assays i.e. Line Blot, Dot blot, have been validated using the IP assay as gold standard, with reported specificity of 96-99% or 98% and sensitivity of 75-93% or 76% respectively. High level of agreement was also found between IP and PMAT (86–88).

Enzyme-linked immunosorbent assays (ELISA) have also been recently developed to detect anti-MDA5 Abs and allow their quantification. An ELISA based on a recombinant MDA5 fusion protein produced in insect cells has shown an analytical sensitivity and specificity of 98% and 100% respectively, compared with IP assays (89). A major benefit of anti-MDA5 Abs ELISA is that it allows a precise quantification of the level of anti-MDA5 Abs in the serum of patients, and a follow-up of its variation during the course of the disease or after the introduction of a therapy.

### Anti-MDA5 Autoantibodies: A Tool for Monitoring Disease Activity

The quantification of anti-MDA5 Abs level seems to be a way to predict disease outcome. When comparing surviving and deceased patients, anti-MDA5 Abs levels are significantly lower in surviving patients. Moreover, the outcome of patients with low titers of anti-MDA5 Abs is approximately the same as patients without anti-MDA5 Abs (90). Anti-MDA5 Abs titers also correlate with the severity of the disease, and more particularly with the severity of ILD and cutaneous symptoms. Several Japanese studies reported higher levels of anti-MDA5 Abs in JDM patients with RP-ILD (63, 64, 67). One study reported deep necrotic ulcerations in the patients with the highest anti-MDA5 Abs levels and only superficial cutaneous symptoms in those with the lowest value (12).

Furthermore, the value of the anti-MDA5 Abs could also be useful for the evaluation of the response to treatment. In a Japanese cohort, patients with anti-MDA5 Abs levels greater than 500 units/mL (positivity threshold at 8 units/mL) were resistant to treatment by glucocorticoids/cyclophosphamide or intravenous immunoglobulins, and died (12). Inversely, patients with anti-MDA5 Abs levels lower than 500 units/mL had less severe lung lesions and cutaneous symptoms improved after treatment. Finally, monitoring the Abs levels along the course of the disease could permit to objective a remission or to detect a relapse early. Remission induces the disappearance of anti-MDA5 Abs, whereas it remains elevated in the patients who die or who later relapse (91, 92).

In conclusion, the anti-MDA5 Abs are a critical diagnostic biomarker of the disease and the level of Abs could be an important prognostic and predictive parameter to monitor in patients.

### Co-Occurrence of Anti-MDA5 Autoantibodies With Other Autoantibodies

Anti-TRIM21 Abs (also known as anti-Ro52/SSA-52 Abs) are nonspecific Abs encountered in several connective diseases, and also frequently detected in the serum of patients with anti-MDA5 DM, with 27 to 62% of dual-positive patients. These patients seem to develop RP-ILD more frequently and have a less favorable prognosis (15, 16, 55, 56).

A study reported the presence of Abs directed against a nuclear protein, the splicing factor proline/glutamine-rich (SFPQ), in the serum of 27 out of 51 patients (53%) with anti-MDA5 DM (93). SFPQ is a multifunctional nuclear protein of 110 kDa, that participates in diverse molecular functions including transcription regulation, and is also involved in the regulation of host innate immune response to viruses (94, 95). Anti-SFPQ Abs have not been detected in other form of DMs, and were identified at diagnosis in 13 patients, while the others turned positive during the disease course (93). Another study observed an apparition of anti-SFPQ Abs at recurrence (47). The clinical relevance of these Abs is unknown, and they are not researched in current practice.

## Pathogenesis of the Disease

Owing to the rarity of anti-MDA5 DM, knowledge on the pathogenic mechanisms of the disease remain limited, but they are believed to occur as a consequence of a particular gene-environmental interaction. Although scarce, several studies have underlined an association of HLA and non-HLA alleles with the disease. Moreover, the identification of seasonal and geographical clustering at disease onset suggests that an infectious agent could be a triggering factor, an attractive hypothesis in view of the antiviral function of the MDA5 antigen. As for the pathogenic mechanisms involved in the disease, since MDA5 is an IFN-I inducible gene, IFN-I could be the starting point for most of the pathophysiological pathways.

### Genetic Susceptibility

HLA allele associations have been described in Asian cohorts. The strongest disease association was found with alleles of the type II HLA alleles HLA-DRB1. Analyses of the relationship between type II HLA alleles and anti-MDA5 DM in Chinese cohorts demonstrated a higher frequency of HLA-DRB1*04:01,*12:02 and *12:01 alleles in Chinese patients with anti-MDA5 DM (96, 97). However, different risk factors are observed in particular ethnic groups, including combined frequency of HLA-DRB1*01:01 and *04:05 in Japanese patients (98), with no significant difference for the same alleles in Chinese populations (96). In addition, some alleles such as HLA-DRB1*09:01 have been associated with a worse prognosis in Chinese patients (97). To date, no association between HLA alleles and anti-MDA5 DM has been identified in Caucasian population (99).

Although HLA allele associations differ across ethnic populations, amino acid sequence variations observed in the type II HLA-DRB1 alleles might affect the structure of the antigen-binding groove of the HLA molecule and thereby influence the antigenic repertoire, increasing by this way the disease susceptibility.

**Figure 3:**
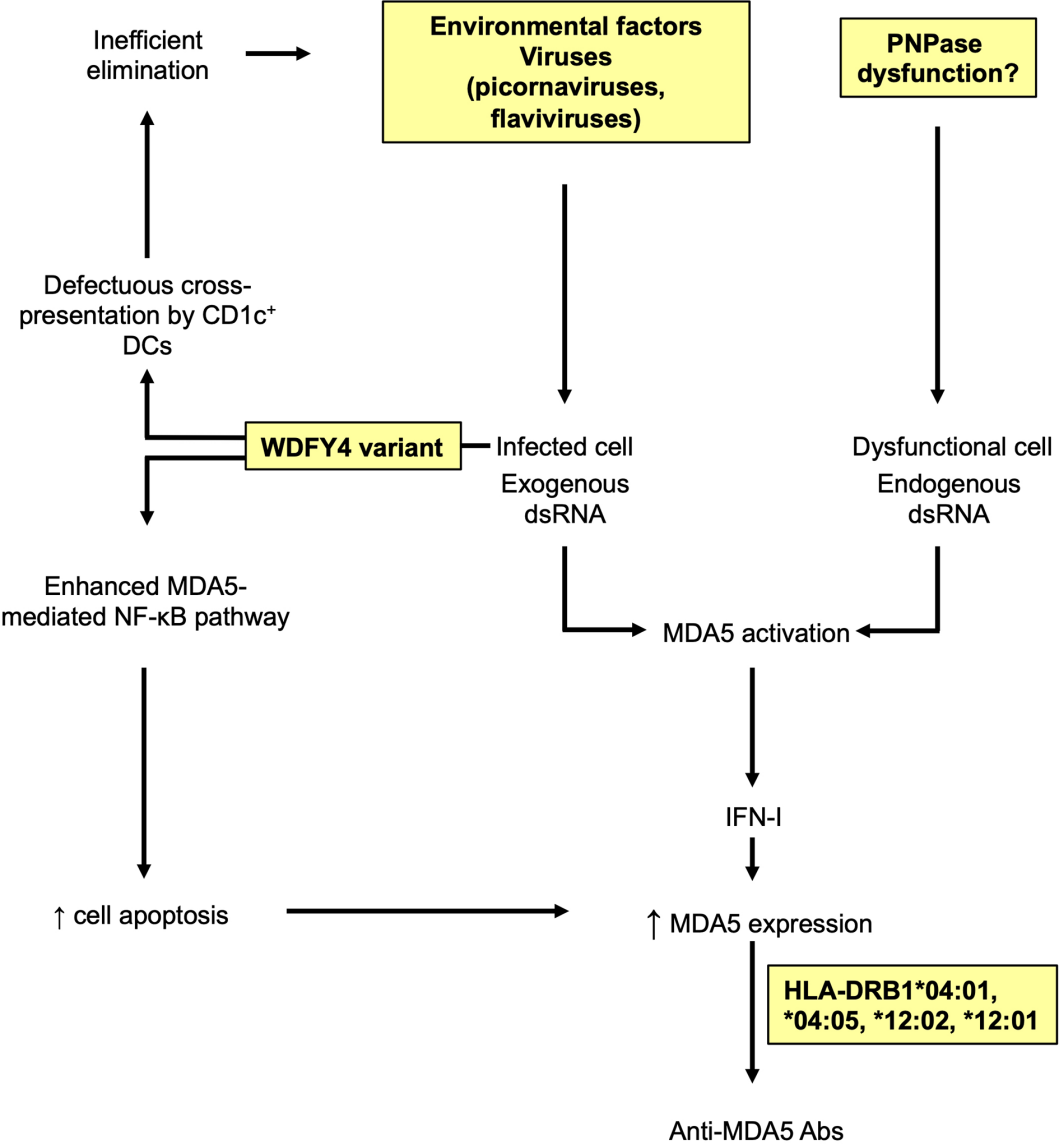
Possible genetic and environmental factors involved in the anti-MDA5 dermatomyositis. Viral double stranded RNA (dsRNA) activates MDA5 in infected cells, leading to type I interferon (IFN-I) production and increased levels of MDA5. Altered WDFY4 impairs antigen cross-presentation by CD1c+ dendritic cells (DCs), favoring an inefficient elimination of infected cells and further activation of MDA5. In parallel, altered WDFY4 also enhances MDA5-mediated nuclear factor-kappa B (NF-κB) pathway leading to the apoptosis of infected cells, and the release of MDA5. Local dysfunctional mitochondrial polynucleotide phosphorylase (PNPase) could lead to intracellular accumulation of endogenous dsRNA, fueling uncontrolled activation and expression of MDA5. Abnormal accumulation of MDA5 may favor a loss of tolerance to MDA5 in an individual with proper genetic background, leading to anti-MDA5 antibodies (Abs).

One non-HLA locus, an intronic variant of WDFY4, has been recently associated with the anti-MDA5 DM in Japanese patients (100). This variant induces a higher expression of a truncated isoform of the WDFY4 protein. Although the precise biological function of WDFY4 was unknown when this work was published, Kochi et al. demonstrated that the truncated form of WDFY4 markedly enhanced the MDA5-mediated NF-κB activation and cell apoptosis. Interestingly, one year later, Theisen et al. uncovered the function of WDFY4, which is in fact a critical regulator of cross-presentation in the conventional CD1c^+^ dendritic cells (101). This is of particular interest in the context of anti-MDA5 DM since, as it will be discussed later, an inadequate immune response to an infectious agent could be the environmental factor triggering the disease. It is then tempting to speculate that qualitative and/or quantitative alterations of WDFY4 might induce a striking defect in cross-presentation of viral-associated antigens, which may trigger secondarily an aberrant autoimmune response (**Figure 3**).

Other molecular defects, unexplored so far, might also be involved in the pathogenesis of anti-MDA5 DM. For instance, given the role of mitochondrial PNPase in eliminating endogenous dsRNA, local dysregulated expression and/or function of the enzyme could generate an accumulation of endogenous dsRNA, leading to MDA5 activation, IFN-I pathway stimulation and elevated MDA5 expression.

### Environmental Factor

The hypothesis of a viral trigger of anti-MDA5 DM is suggested by epidemiological studies that highlighted a seasonal distribution of the disease. Classically, anti-MDA5 DM onset follows a seasonal repartition with an epidemic period in fall and winter, a peak in late winter and spring and a dip in summer, following respiratory virus epidemic period (102, 103). In the hypothesis of a viral infection as a possible initiator of autoimmunity, the scenario could involve an activation of MDA5 in the infected cells, leading to IFN-I production and increased levels of MDA5, followed by an excessive local apoptosis favored by a specific genetic background (intronic variant of WDFY4). The release of the MDA5 antigen into the microenvironment following cell lysis could be the cause of a loss of tolerance towards MDA5, resulting in the production of anti-MDA5 Abs. This mechanism could also be fueled by a defect in the elimination of the virus, due to an inefficient cross-presentation of viral antigens by CD1c^+^ DCs (**Figure 3**).

It is also interesting to note that a geographical distribution of the disease has also been reported in Japan, with an increased prevalence of anti-MDA5 DM in patients living in rural areas as opposed to urban areas as well as in patients residing near freshwater (103, 104).

### Pathogenesis of the Anti-MDA5 Dermatomyositis: The Vasculopathy Hypothesis

Several pieces of evidence point toward a central role of vascular injury in the pathogenesis of anti-MDA5 DM: (i) Patients frequently show typical cutaneous features such as skin ulcers due to severe vasculopathy; (ii) Histopathology of palmar papules, as well as lung tissues highlights vasculopathy (12, 15, 105); (iii) Biomarkers of endothelial dysfunctions have been identified in the sera of patients (106, 107); (iv) Patients exhibited a strong IFN-I signature distributed in the vasculature of the skin (108, 109). It is important to emphasize that these particular features are observed in the skin and the lungs of patients with anti-MDA5 DM but that there is no or few signs of vasculopathy nor of an enhanced IFN-I signature, compared to other DM, in muscle tissue of anti-MDA5 DM patients (21, 22).

Biomarkers of endothelial dysfunctions released in the sera of anti-MDA5 DM patients include endothelin, thrombomodulin, plasminogen activator inhibitor, von Willebrand factor (vWF), soluble vascular cell adhesion molecule-1 and soluble intercellular adhesion molecule-1 (106, 107). Interestingly, serum levels of endothelin and vWF are higher in anti-MDA5 DM patients who have cutaneous ulcers and ILD and IFN-I signature correlates positively with endothelin levels (106). All together, these data suggest that blood vessels exposure to IFN-I in anti-MDA5 DM may lead to endothelial injury, ultimately responsible for the cutaneous and pulmonary lesions associated with this disease.

#### Vasculopathy and Pulmonary Lesions

**Figure 4:**
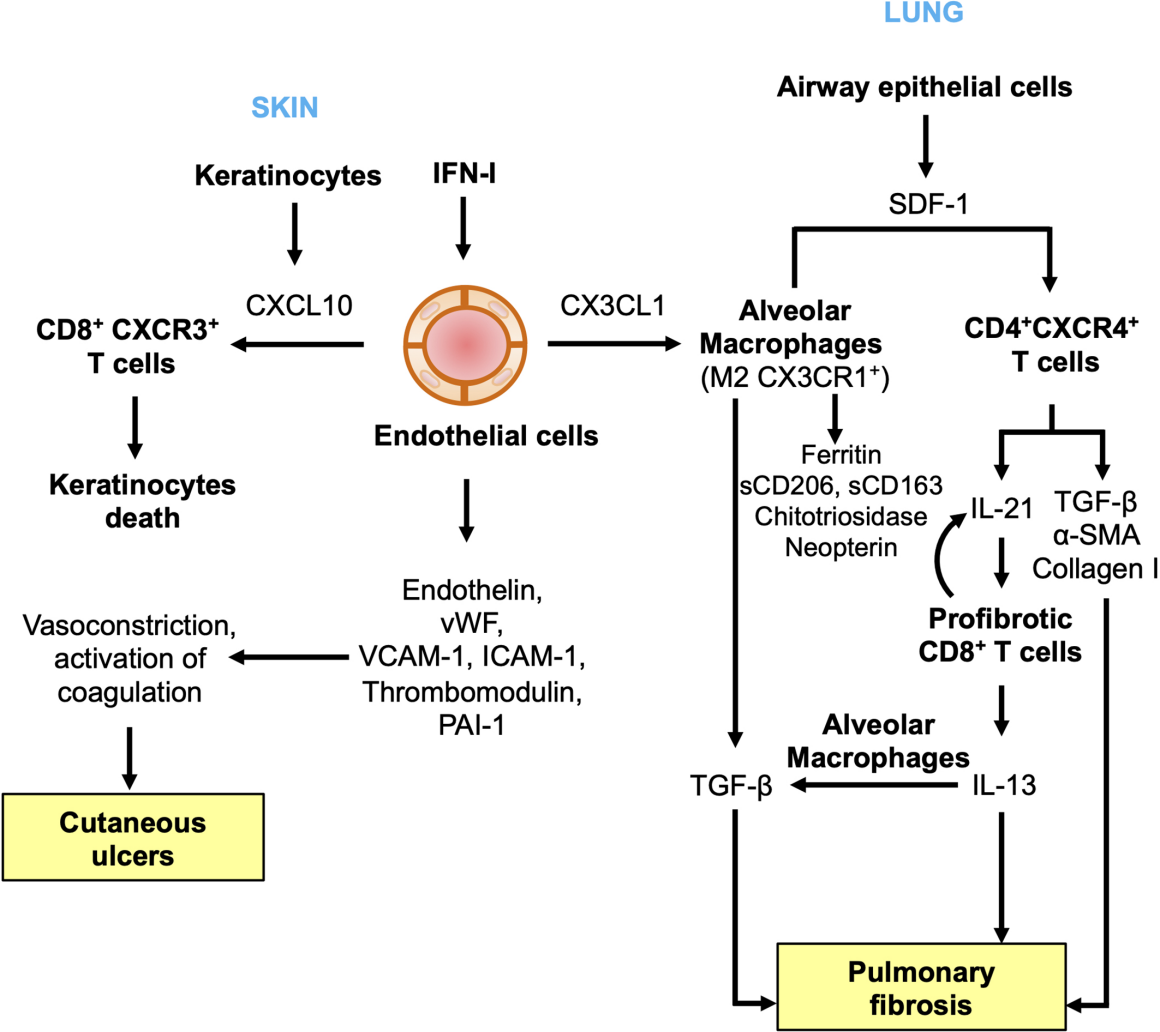
Endothelial dysfunctions and immune alterations in anti-MDA5 dermatomyositis. In the skin, CXCL10, a keratinocyte and endothelial-derived chemokine, induces the recruitment of CD8^+^CXCR3^+^ T cells, potentially autoreactive and leading to keratinocytes death. Endothelin released by injured endothelial cells, is a strong vasoconstrictor which can induce local ischemia responsible for cutaneous ulcers. In the lung, CX3CL1 can be produced by vascular pulmonary endothelial cells following IFN-I exposure. CX3CL1 recruits CX3CR1^+^ alternative alveolar macrophages (M2). Alveolar M2 macrophages, as well as airway epithelial cells, release stromal cell-derived factor 1 (SDF-1) which induces the accumulation of intrapulmonary CD4^+^CXCR4^+^ T cells. CD4^+^CXCR4^+^ T cells produce profibrotic agents (transformation growth factor-β (TGF-β), α-smooth muscle actin (α-SMA) and collagen I), as well as IL-21, which promotes the differentiation of profibrotic CD8^+^ T cells. CD8^+^ T cells secrete IL-13, which stimulates macrophages to produce profibrotic factors.

Anti-MDA5 DM is associated with an accumulation of activated macrophages (M2) in alveoli (110, 111). Several markers of macrophage activation are elevated in the serum of anti-MDA5 DM patients, such as a soluble form of CD206, which is highly expressed by alveolar macrophages (110, 112). Other soluble markers of activated macrophages, such as a soluble form of CD163, as well as the chitotriosidase and the ferritin, are also found in the sera of patients at significantly higher levels than in other subtypes of DM (110, 111, 113, 114). Neopterin levels, another marker of macrophage activation, are elevated in DM patients with anti-MDA5 Abs in association with RP-ILD and reduced survival. A positive correlation of neopterin levels with ferritin and markers of disease severity, and a negative correlation with pulmonary function have been demonstrated (115). Multiple chemokines can induce the recruitment of M2 macrophages. Among them, the chemokine CX3CL1 (Fractalkine) is of interest in anti-MDA5 DM since its concentration is elevated in sera of patients (116). CX3CL1 secretion can be induced by IFN-I in pulmonary vascular endothelial cells and induces the recruitment of CX3CR1^+^M2 macrophages in the lungs (117, 118). It is then tempting to speculate that high levels of IFN-I induce endothelial injury leading to the secretion of CX3CL1, responsible for the recruitment of intrapulmonary profibrotic M2 macrophages. Locally, M2 macrophages produce TGF-β to directly promote pulmonary fibrosis, and could be involved in the recruitment of profibrotic CD4^+^CXCR4^+^ T cells. In agreement with this, elevated levels of CD4^+^CXCR4^+^ T cells are observed in the peripheral blood and the bronchoalveolar lavage fluids of patients with ILD. Locally, the airway epithelial cells and the macrophages are the main sources of the stromal cell derived factor-1 (SDF-1 or CXCL12), the ligand of CXCR4 (119). Once recruited, the CD4^+^CXCR4^+^ T cells may promote pulmonary fibroblast proliferation, partly through the release of IL-21, and secondarily by the production of profibrotic agents, namely TGF-β, α-smooth muscle actin and collagen I. IL-21 is known to induce the differentiation of IL-13 producing-CD8^+^ T cells, which in turn enhance IL-21 production, creating an activation loop (120). IL-13 may fuel pulmonary fibrosis through two mechanisms: direct activation of fibroblast, and stimulation of the synthesis of TGF-β by activated macrophages (121) (**Figure 4**).

#### Vasculopathy and Cutaneous Lesions

Histology of skin biopsies from patients with anti-MDA5 DM classically show an interface dermatitis, a histopathological pattern characterized by vacuolar changes, apoptotic keratinocytes and infiltration of CD8^+^ lymphocytes at the dermoepidermal junction (122–124). The interface dermatitis is also a classical feature observed in systemic erythematosus lupus and lichen planus. Another common feature of all these diseases is the enhanced IFN-I signaling into the skin of patients. A common pathophysiological model has therefore been proposed, based on the secretion of keratinocyte-derived CXCL10 following skin exposure to IFN-I (125–128). Consistent with this observation, the expression of CXCL10 as well as the expression of IFN-I induced proteins (ISG15, MxA) are upregulated in the skin of anti-MDA5 Abs positive patients (108, 109, 126, 129). Endothelial cells stimulated by IFN-I could also constitute another source of CXCL10 (130). CXCL10 then induces the recruitment of CXCR3^+^CD8^+^ T cells, potentially autoreactive, responsible for the apoptosis of keratinocytes (125, 126) (**Figure 4**).

In addition, skin biopsies from anti-MDA5 DM patients also show more severe lesions, affecting the deeper layers of skin, down to the dermis. Epidermal necrosis and typical findings of vasculopathy are observed, with vascular fibrin deposition, thickening of the vascular walls and vascular thrombosis of the small and medium vessels (12, 15, 123, 131). Endothelin, released consequently to endothelial injury caused by chronic exposure to IFN-I, acts as a potent vasoconstrictor. It may in part be responsible for the skin ulcers, by inducing a local ischemia, as is the case in other diseases (132). The regression of skin ulcers in patients treated with bosentan, an endothelin-receptor inhibitor, or with a vasodilator drug, such as sildenafil, supports this hypothesis (133–135). In the same way, pro-coagulant factors (e.g. vWF) released by damaged endothelial cell could lead to blood coagulation activation, as evidenced by the vascular fibrin deposits and thromboses observed histologically (**Figure 4**).

### Potential Contribution of Anti-MDA5 Antibodies to the Pathogenesis

Whether we consider skin or lung lesions, IFN-I seems to be the starting point for all the pathophysiological pathways described above. Indeed, IFN-I signaling is enhanced in anti-MDA5 DM, in skin and in serum, more than in other DM, suggesting the presence of a specific trigger of the IFN-I pathway in the disease (109, 129, 136). Here we assume that anti-MDA5 Abs could be the cornerstone of the dysregulation of the IFN-I pathway in anti-MDA5 DM. Several observations support this hypothesis: (i) The severity of the disease correlates with the titers of anti-MDA5 Abs (12, 63, 64, 67); (ii) The use of therapies that target the humoral immune response has shown its effectiveness in patients with anti-MDA5 DM (137–141); (iii) MDA5 expression has been shown to be enhanced in skin biopsies of patients (79).

**Figure 5:**
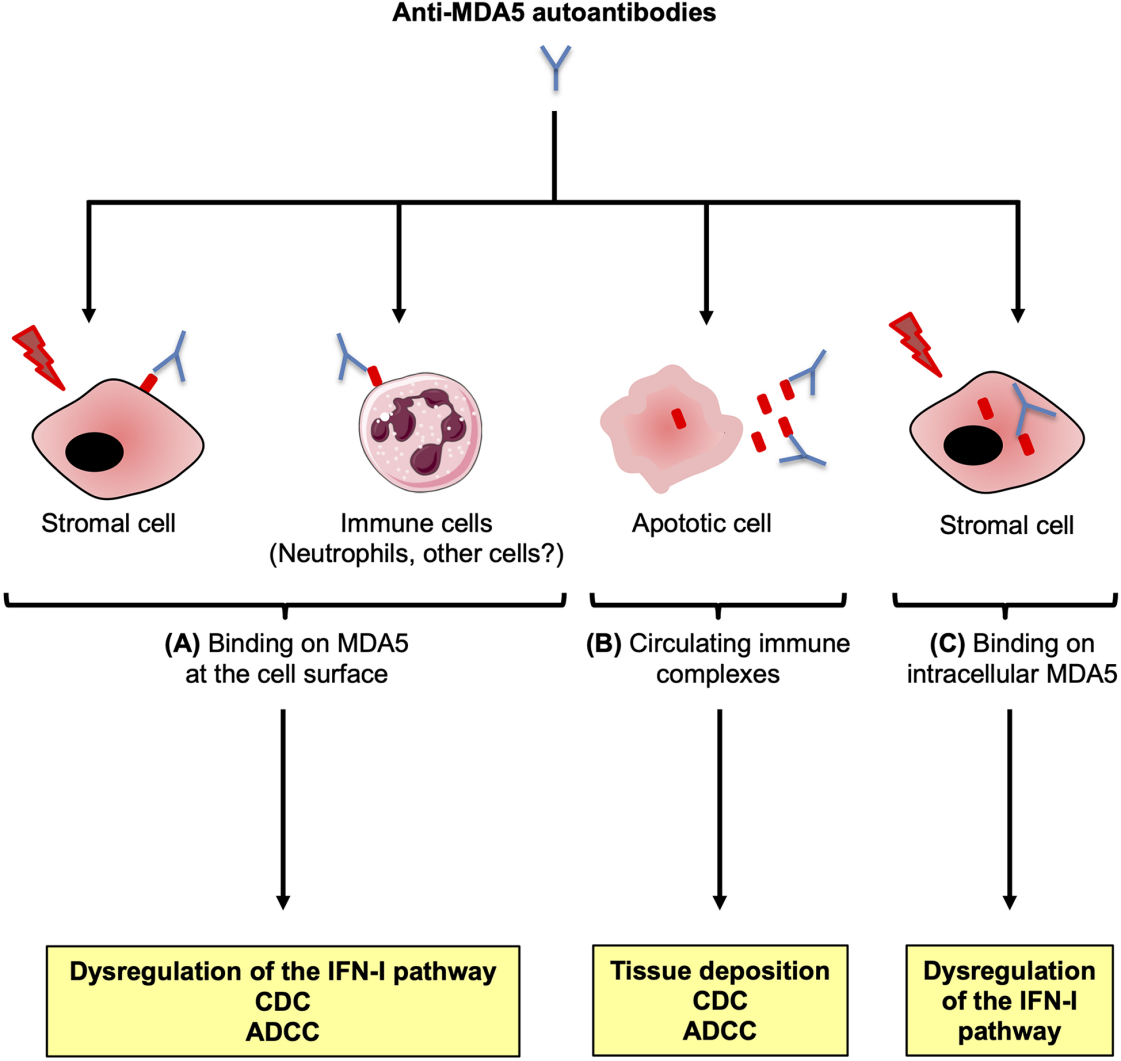
Potential contribution of the anti-MDA5 antibodies to the pathogenesis. Anti-MDA5 antibodies (Abs) may contribute to the pathogenesis in several ways. **(A)** In specific conditions, MDA5 may translocate at the surface of critical stromal cells, or immune cells such as neutrophils. Interaction between the autoAb and the ectopic antigenic target could trigger chronic activation of the type I interferon (IFN-I) signaling pathway, as well as immune mediated cytotoxicity through complement activation (CDC) and/or Ab-dependent cytotoxicity (ADCC). **(B)** Anti-MDA5 Abs could also bind to MDA5 released from apoptotic cells, to form immune complexes that could contribute to immune-mediated damage. **(C)** A cell exposed to a stress (infection, genetic background) overexpresses intracytoplasmic MDA5. Anti-MDA5 Abs might penetrate the cell to bind to MDA5, altering several functional pathways.

In pathological contexts, MDA5 is overexpressed in altered tissues such as the skin of patients and ectopic expression of MDA5 at the cell surface has been reported (79, 80). In this context, anti-MDA5 Abs could then bind to MDA5-positive cells and induce an inappropriate activation of MDA5, leading to the dysregulation and the chronic activation of the IFN-I pathway in target tissues, worsening the existent lesions. Anti-MDA5 Abs binding to its antigenic target could also induce immune-mediated cytotoxicity by complement fixation or antibody-dependent cytotoxicity, further worsening the lesions (**Figure 5A**). Apart from binding to MDA5 expressed on cell surface, anti-MDA5 Abs could also form immune complexes with the MDA5 proteins released from apoptotic skin and/or lung fibroblasts. These immune complexes could then deposit in organs, for example in dermal/lung vessels, inducing more vascular damage (**Figure 5B**). Finally, anti-MDA5 Abs could penetrate cells and interact with cytoplasmic MDA5, similarly to what has been described with other Abs, altering several functional pathways (**Figure 5C**) (142–144).

The isotype and the subclass of anti-MDA5 Abs might also affect their potential pathogenic function. In a Chinese cohort, anti-MDA5 IgA and IgG were the predominant isotypes. Interestingly, anti-MDA5 IgG1 were associated with higher serum ferritin levels, severe interstitial pneumonia, and a higher mortality rate. The combined positivity for anti-MDA5 IgG1 and anti-MDA5 IgG4 was predictive of poor prognosis (145). IgG1 are potent activators of the complement pathway, and as a result, anti-MDA5 IgG1 could be the main subclass involved in the pathogenesis of the disease. The identification of anti-MDA5 IgG4 is more unexpected, as IgG4 are considered non-inflammatory, owing to the unique structure of their hinge region (146). Whether the presence of anti-MDA5 IgG4 in the most severe forms of the disease reflects a modulatory mechanism, although insufficient, remains to be defined.

### Similarities Between Anti-MDA5 Dermatomyositis and COVID-19

Anti-MDA5 DM and coronavirus disease 2019 (COVID-19) share several common features, clinical and pathogenic, and exploring the pathophysiological mechanisms of COVID-19 may help to better understand the pathogenesis of anti-MDA5 DM (147).

First, as for anti-MDA5 DM, COVID-19 can be complicated by interstitial pneumonia which can lead to acute respiratory distress syndrome and death. This pulmonary damage is difficult to distinguish from the ILD associated with anti-MDA5 DM (148). Thus, it is important to carefully assess patients with RP-ILD, in particular in the case of a negative RT-PCR test for SARS-CoV-2 (severe acute respiratory syndrome coronavirus 2) as RP-ILD may be the sole symptom at anti-MDA5 CADM onset. Anti-MDA5 DM may then be easily confounded with COVID-19, delaying the diagnosis and treatment. Inversely, patients with positive test for anti-MDA5 Abs, or any other MSA, and who develop acute pneumonia should be tested for SARS-CoV-2 infection, as the infection worsens the patient’s condition (149).

Second, anti-MDA5 DM and COVID-19 seem to share several pathogenic mechanisms. Both diseases are characterized by an activation of the IFN-I signaling pathway. However, although in anti-MDA5 DM, dysregulation of the IFN-I axis seems to exacerbate the disease, the role of the IFN-I pathway in COVID-19 appears more complex. MDA5 is a sensor of SARS-CoV-2 in lung cells, and its activation induces an IFN response to eliminate the virus (150–152). The IFN-I pathway plays a major role in the defense against the virus in COVID-19, as illustrated by the impaired IFN-I signaling in severe cases and the presence of anti-IFN-I Abs in the sera of more than 10% of patients with a life-threatening infection (153–156). In this sense, early activation of the IFN-I signaling pathway appears to be an essential weapon against SARS-CoV-2 infection. In contrast, a strong delayed IFN-I response could exacerbate the hyperinflammation associated with the most severe forms of COVID-19 (157, 158).

Another common pathological feature shared by the two diseases is the endothelial injury and the thrombotic manifestations associated with the more severe forms (148, 159, 160). This is highlighted by the presence of markers of endothelial and platelet activation as well as coagulation factors and fibrinolytic enzymes in the serum of patients, such as vWF, thrombomodulin, P-selectin and D-dimer (161). The important systemic inflammatory response and activation of the angiotensin converting enzyme 2 (ACE2), the major cell entry receptor for SARS-CoV-2, expressed on endothelial cells are accountable for these vascular damages (159, 162).

Finally, anti-MDA5 Abs have been identified in the serum of Chinese patients diagnosed with COVID-19 (48.2% of 274 patients), frequently in the most severe cases. Furthermore, the titers of anti-MDA5 Abs appear to correlate with the severity of the disease and were higher in the non-survival cases (163). These preliminary results should, however, be taken with caution. Indeed, although the titers of anti-MDA5 Abs are statistically higher in the non-survivals infected SARS-CoV-2 patients *versus* the survivals, the orders of magnitude are very low (5.95 ± 5.16 U/mL *vs* 8.22 ± 6.64 U/mL, P = 0.030). In addition, it is well established that common viral infections, as well as SARS-CoV-2 infection, frequently trigger the induction of transient, low-titer, polyspecific autoantibodies (164, 165). In agreement with this, several myositis-specific autoantibodies have been identified as false positives in patients with COVID (166). To what extent these anti-MDA5 Abs are really specific, to what extent they are present in the infection of other RNA viruses or only during the infection by SARS-CoV-2, and what are their implications for the patient in the short and long term are all unanswered questions to be explored.

## Treatment of Anti-MDA5 Dermatomyositis

Although no recommendations for the management of anti-MDA5 DM exist at this time, a combination immunosuppressive (IS) therapy is wildly used. This intensive IS treatment classically consists of an association of glucocorticoids with a calcineurin inhibitor (cyclosporine A or tacrolimus) or a triple therapy with the addition of intravenous cyclophosphamide or mycophenolate mofetil. However, many cases are refractory to this treatment with a reported overall mortality rate after treatment of 40% (167). Resistance to treatment, beyond worsening clinical signs, can be assessed by monitoring the level of anti-MDA5 Abs, ferritin or IL-18 which tend to stabilize or increase in patients refractory to treatment (51, 91, 168, 169). Other studies evaluating the effectiveness of intensive IS therapy highlighted the importance of beginning the treatment early after the diagnosis to improve the prognosis (91, 170). In addition to being partially efficient, the intense IS bi- or tri-therapy is responsible for several adverse events, mostly infections and renal function alteration. Renal function alteration is mostly due to calcineurin inhibitors and lead to treatment interruption in most cases. Bacterial, viral and fungal infections are reported, the most frequent being the reactivation of cytomegalovirus (29, 46, 171, 172). *Pneumocystis jirovecii* pneumonia is also encountered (29, 173, 174). Whether the infections are the result of IS treatment or of the disease itself is not clear, although DM was associated with a higher risk of developing pneumocystosis in a French retrospective study (175).

Due to the limited effectiveness and frequent adverse events of these treatments, several alternative therapy strategies are currently evaluated to treat refractory cases. The rare studies evaluating alternative therapies, such as the use of vasodilators, inhibitors of the IFN axis, or therapies that target the humoral immune response, illustrate the importance of the dysregulated pathways discussed previously in the pathogenesis of the disease.

Data regarding the improvement of cutaneous lesions after intensive IS treatment are scarce but some case reports of cutaneous lesions refractory to IS drugs have been published (48, 137, 176). As the vascular injury seems to have a central role in the development of skin lesions, drugs such as sildenafil, a vasodilatator, or bosentan, an endothelin-receptor inhibitor, might be added to the IS treatment to treat the skin ulcerations (32, 133–135).

Considering the importance of the IFN-I signaling pathway in anti-MDA5 DM, inhibitors of Janus Kinases (JAK) appear to be a promising treatment for anti-MDA5 DM. Several studies reported the efficacy of tofacitinib and ruxolitinib in patients who experienced a relapse or were refractory to standard treatment (50, 176–179). JAK/STAT signaling pathway is activated by IFNs leading to the transcription of IFN-stimulated genes (ISGs), including MDA5. Tofacitinib and ruxolitinib inhibit this pathway, decreasing MDA5 expression and activation. Furthermore, its effectiveness on refractory forms of ILD reinforces the potential role of IL-21 in the pathophysiology of ILD as JAK-pathway is required for IL-21/IL-21R signaling (119, 180, 181). JAK inhibitors could then be used to treat or prevent severe forms of ILD. Tofacitinib seems well tolerated in most patients but one study reported cytomegalovirus reactivation in 100% of patients, varicella-zoster virus reactivation (60%) and bacterial respiratory infections (80%) (178). Whether these adverse events were caused by the disease or the therapy was not determined. The question remains whether JAK-inhibitors should be administered in patients refractory to classic IS therapy or be initiated at diagnosis to avoid worsening of the disease. A clinical trial including 50 Japanese patients with anti-MDA5 CADM-associated ILD diagnosed for less than 3 months reported a 6-month survival significantly higher (100%) in the group of patients (n=18) who received a glucocorticoid combined with tofacitinb than in the group (n=32) who received conventional immunosuppressive treatment (6-month survival of 78%) (182). JAK inhibitors could therefore have a prominent place in the first-line treatment of anti-MDA5 DM.

A pathogenic role of anti-MDA5 Abs could motivate the use of plasmapheresis, IV immunoglobulins and rituximab. Although evidence is limited to small case series, these therapies seem efficacious (137–141, 183–187), apart from one case report of a patient whose condition worsened after plasma exchange probably due to transfusion-related acute lung injury (188). However, their effectiveness in large cohorts of patients as well as data concerning long-term remission remain to be evaluated.

## Conclusion

Anti-MDA5 DM is a systemic autoimmune disease that can be divided into 3 clinical subgroups, with different prognosis, linked to the incidence of RP-ILD which is influenced by the ethnic origin of the patients. Recent publications suggest a central role for IFN-I mediated vasculopathy. It might be responsible for both the pulmonary and the cutaneous lesions, through the secretion of endothelial-derived substances inducing the recruitment and the activation of immune cells, *in fine* responsible for the lesions observed in anti-MDA5 DM. Anti-MDA5 Abs might also contribute to the pathogenesis by altering the IFN-I pathway. Of course, further studies need to be conducted to confirm these assumptions. Elucidating the precise role of anti-MDA5 Abs associated to the disease will constitute a crucial step in the understanding of the pathogenesis. An improved knowledge of the pathogenesis of the disease will also undoubtedly pave the way for the development of more effective therapeutic strategies.

## Author Contributions

AN and FC: writing and figures. FC and NF: concept and proof reading. All authors contributed to the article and approved the submitted version.

## Funding

This work was supported by Hospices Civils of Lyon, the Foundation Arthritis and the association “Les Eclaireuses”.

## Conflict of Interest

The authors declare that the research was conducted in the absence of any commercial or financial relationships that could be construed as a potential conflict of interest.

## Publisher’s Note

All claims expressed in this article are solely those of the authors and do not necessarily represent those of their affiliated organizations, or those of the publisher, the editors and the reviewers. Any product that may be evaluated in this article, or claim that may be made by its manufacturer, is not guaranteed or endorsed by the publisher.
